# Metabolizable protein requirements for maintenance and growth in female Saanen goats approaching maturity

**DOI:** 10.3168/jdsc.2026-1087

**Published:** 2026-09-18

**Authors:** Pedro A. Sampaio, Queila A. Alves, Gabriel M. Moreira, Fernanda O.M. Figueiredo, Bruno Biagioli, Kleber T. Resende, Izabelle A.M.A. Teixeira

**Affiliations:** 1 Department of Animal, Veterinary and Food Sciences, University of Idaho, Twin Falls, ID 83303-1827; 2 Department of Animal Science, UNESP (Universidade Estadual Paulista), Jaboticabal, SP, Brazil 14884-900

## Abstract

Estimating protein requirements is essential to improve N utilization in dairy goats, benefiting economic efficiency and environmental stewardship. This study aimed to estimate MP requirements for maintenance and growth in female Saanen goats weighing 30 to 45 kg. Data were collected from 2 comparative slaughter experiments. In the first experiment, 24 goats with an initial BW of 30 kg were used to estimate MP requirements for maintenance (MPm). Six goats were slaughtered at the beginning of the experiment to determine baseline body composition. The remaining goats were assigned to 3 intake levels (ad libitum, 30% restriction, and 60% restriction). The MPm was estimated from the regression of retained protein on MP intake (MPI). The MPm was 2.5 g/kg BW^0.75^ per day. In the second experiment, 18 goats with an initial BW of 30 kg were used to estimate protein requirements for growth. Goats were fed ad libitum and slaughtered at 30, 38, and 45 kg of BW. The MP requirements for growth (MPg) were derived by dividing net protein requirement for growth (NPg) by the efficiency of metabolizable protein use for growth (K_PG_), resulting in MPg ranging from 396.73 to 394.89 g/kg BW gain as BW increased from 30 to 45 kg. Our findings provide refined estimates of MP requirements for female Saanen goats approaching maturity and support improved precision in diet formulation and N utilization efficiency in dairy goat production systems.

Optimizing protein nutrition is essential for improving N utilization and overall sustainability in dairy goat production systems. In ruminants, MP represents the true protein (i.e., AA) absorbed in the small intestine, derived from microbial protein and RUP (NRC, 2007), and accurate estimation of MP requirements is critical for balancing productivity with environmental impact. Overfeeding protein increases feed costs and N excretion, negatively affecting the environment, whereas underfeeding limits animal performance, emphasizing the need for precise protein supply during key physiological stages.

Although Saanen goats are among the most widely used dairy breeds worldwide, information on their MP requirements remains limited, particularly for growing female goats. Defining these requirements is fundamental for developing cost-effective and environmentally responsible feeding strategies. For instance, Härter et al. (2017) evaluated MP requirements during pregnancy in multiparous goats (i.e., mature goats), Luo et al. (2004) synthesized data across multiple breeds, categories, and BW, and Souza et al. (2017, 2021) evaluated sex effects on protein requirements in growing goats using a meta-analytical approach. However, these studies generally encompass heterogeneous populations or rely on diverse methodological approaches, which may limit the precision of requirement estimates for specific physiological stages.

The BW range from 30 to 45 kg represents a critical phase in the development of female dairy goats, comprising the transition from the prebreeding period to late growth. Female goats are generally recommended for first breeding after reaching at least 70% of their mature BW. In large dairy goat breeds, this target is typically achieved at approximately 35 to 45 kg BW (Greyling, 2010). Based on the maturity estimates proposed by Almeida et al. (2016), who defined mature empty body weight (**EBW**) using body composition criteria, female goats weighing 30, 35, 40, and 45 kg of BW represent approximately 92%, 111%, 130%, and 149% of the estimated mature EBW, respectively. Therefore, the evaluated BW range spans goats approaching the stage at which protein accretion stabilizes, as well as animals that have largely completed this phase of body protein deposition. During this period, the composition of weight gain progressively shifts from lean tissue toward fat deposition, resulting in reduced protein accretion and lower MP requirements for growth, whereas maintenance accounts for an increasing proportion of the total MP requirement. Nutritional management during this period remains important because it can influence growth efficiency, body development, and subsequent milk production potential (Souza et al., 2020; Pilajun et al., 2023). Despite its biological and practical importance, MP requirements for female Saanen goats within this developmental stage remain insufficiently defined.

We hypothesized that late growth in female Saanen goats is characterized by changes in body composition that require stage-specific estimates of MP requirements for maintenance and growth. Therefore, the objective of this study was to estimate MP requirements for maintenance and growth in female Saanen goats weighing 30 to 45 kg using the comparative slaughter technique. These estimates aim to support more precise diet formulation and improved N utilization efficiency in dairy goat production systems.

Two experiments were carried out at the Goat Facility of the Universidade Estadual Paulista (UNESP, Jaboticabal, São Paulo, Brazil) to estimate MP requirements for maintenance and growth in growing female Saanen goats using the comparative slaughter technique. All experimental procedures were approved by the Institutional Animal Care and Use Committee of UNESP (protocol #008919-08).

General experimental procedures employed in this study are described in Figueiredo et al. (2016). In brief, rations were formulated to meet the nutritional requirements of growing female dairy goats, according to NRC (2007), and consisted (on DM basis) of Tifton hay (24.7%) and a concentrate mixture containing cracked corn grain (50.0%), soybean meal (13.5%), wheat bran (9.0%), limestone (1.6%), sodium bicarbonate (0.3%), mineral premix (0.3%), ammonium chloride (0.1%), and salt (0.3%). The TMR contained (% of DM, unless otherwise noted) 13.6% CP, 2.8% ether extract, 31.4% NDF, 13.2% ADF, 2.76% ADL, 6.24% ash, 93.8% OM, 45.9% NFC, and 39.6% starch, with 1.73 Mcal ME/kg DM. The RDP and RUP accounted for 62.7% and 37.3% of CP, respectively. The ration was offered twice daily. Individual feed intake was recorded daily based on feed offered and refusals. Once goats reached the predetermined BW for slaughter, they underwent a 16-h fasting period (solid feed only). After fasting, they were stunned and slaughtered by severing the jugular veins and carotid arteries. Blood was collected and weighed. Skin was removed, weighed, and sampled from multiple locations for further analysis. The gastrointestinal tract (**GIT**) was weighed before and after the removal of its contents. The weight of the GIT contents was used to calculate the EBW by subtracting the contents of GIT and bladder from the slaughter BW. The entire body of each animal, including blood, organs, head, feet, and carcass, was frozen at −20°C, then processed using a band saw, ground, and homogenized. Three representative samples were taken from the homogenized whole body. Frozen skin samples were ground and homogenized separately. Body and skin samples were stored frozen for subsequent laboratory analysis. Further details are available in Figueiredo et al. (2016).

In the first experiment, 24 female Saanen goats (initial BW = 30 ± 0.81 kg; initial age: 7 mo) were used to estimate protein requirements for maintenance. Six goats were slaughtered at the beginning of the experiment to estimate the initial body composition (baseline animals). The remaining goats were randomly assigned to 6 blocks of 3 goats each, with intake levels treatments (ad libitum, 30% restriction, and 60% restriction) randomly assigned within each block. The feed intake of the ad libitum-fed goat in each block determined the feed allowance for the restricted-fed goats (pair-feeding). The entire block was slaughtered when the ad libitum-fed goat reached 45 kg of BW. The average experimental period was 181 ± 25 d.

A linear regression of retained total body N (g N/kg BW^0.75^ per day) on CP intake (g CP/kg BW^0.75^ per day) was used to estimate the net protein requirements for maintenance (**NPm**). The intercept of this regression was assumed to represent the endogenous and metabolic losses of N. Multiplying this intercept by 6.25 (the conversion factor from N to protein) yields NPm.

Metabolizable protein requirement for maintenance (**MPm**) was estimated by regressing retained protein on MP intake (**MPI**). The MPI was calculated as the sum of truly digestible microbial crude protein (**MCP**) and the digestible RUP intake. The MCP synthesis was estimated using the equation proposed by Santos et al. (2021): MCP (g/d) = 12.73 + 15.30 × MEI (Mcal/d), where MCP is the daily microbial protein synthesis (g/d) and MEI is the ME intake (Mcal/d). Metabolizable energy intake (**MEI**) was calculated as the difference between gross energy intake and total energy losses in feces, urine, and gases (Souza et al., 2020). The truly digestible fraction of MCP was assumed to be 80% (NRC, 2000). The RUP intake was estimated using NRC (2000) feed composition library, assuming intestinal digestibility of 80% (NRC, 2000). The MPm was determined by setting retained protein to zero. The efficiency of MP use for maintenance (**K_PM_**) was calculated as NPm/MPm.

In the second experiment, a total of 18 female Saanen goats (initial BW = 30 kg and initial age = 7 mo) were used in a completely randomized design to estimate protein requirements for growth. Goats were fed ad libitum and slaughtered at a target BW of 30, 38, and 45 kg.

Protein body composition was determined by analyzing the protein content of the whole empty body. The protein content of female goats was summarized using the following allometric equation (Equation 1):

[1] Log_10_Protein = β0 + β1 × Log_10_EBW,

where Log_10_Protein is the log-transformed total amount of protein in the empty body (g), Log_10_ EBW is the log-transformed empty body weight (kg), and β0 and β1 are the regression parameters. The first derivative of Equation 1 was used to estimate the net protein requirement for growth (**NPg**; Equation 2):

[2] NPg = β1 × 10^β0^ × EBW^(β1−1)^,

where NPg is the net protein requirement for growth (g/kg EBW gain), β0 and β1 are the regression parameters of Equation 1, and EBW is the empty body weight (kg).

The metabolizable protein requirement for growth (**MPg**) was estimated by dividing NPg by the efficiency of metabolizable protein use for growth (**K_PG_**).

The K_PG_ was estimated by regressing retained protein (g/d), calculated using the comparative slaughter technique, on daily MPI above maintenance. For this calculation, we used the MPm estimated in this study (experiment 1). This regression was constrained to have an intercept equal to 0.

All statistical analyses were performed using the R software (version 4.1.1, R Core Team, 2021-08-10).

Data from the first experiment were analyzed as a randomized block design, considering intake levels as a fixed effect and blocks as a random effect. The second experiment was analyzed as a completely randomized design, considering slaughter weight as a fixed effect. Model assumptions (i.e., homoscedasticity and normality) were evaluated graphically and outliers were defined when the standardized residual was outside |3|. Linear regression analyses in both experiments were performed using the lme function in R.

After parameter estimation, Monte Carlo simulations (10,000 iterations) were performed to derive 95% CI for all protein requirements estimates. For each BW, simulated values of NPm, MPm, NPg, and MPg were generated assuming a multivariate normal distribution of model parameters and their associated error terms, using the algorithm described by Fan et al. (2002).

Feed restriction generated a wide range of nutrient intake, growth performance, and protein retention, providing the variation required to estimate NPm and MPm. The goats slaughtered at the beginning of the experiment (baseline) had a BW of 30.10 ± 0.53 kg and an EBW of 22.34 ± 1.06 kg. Goats assigned to the ad libitum, 30% feed restriction, and 60% feed restriction had initial BW of 29.83 ± 0.26, 29.58 ± 1.34, and 28.20 ± 0.40 kg and reached final BW of 45.50 ± 0.35, 35.40 ± 1.52, and 27.02 ± 1.58 kg (EBW: 39.48 ± 0.70, 30.88 ± 2.21, and 21.83 ± 1.51 kg), corresponding to ADG of 88.26 ± 26.73, 30.89 ± 14.66, and −5.53 ± 9.14 g/d, respectively. Daily DMI decreased accordingly, averaging 1.07 ± 0.21, 0.68 ± 0.12, and 0.37 ± 0.07 kg for ad libitum, 30% feed restriction, and 60% feed restriction, with corresponding CP intake of 154.58 ± 33.87, 99.85 ± 18.87, and 55.00 ± 9.81 g/d and MEI of 2.24 ± 0.40, 1.59 ± 0.22, and 0.86 ± 0.31 Mcal/d. The resulting MPI, calculated as previously described, averaged 84.07 ± 13.14, 59.08 ± 7.86, and 36.98 ± 6.73 g/d, resulting in retained protein of 12.13 ± 2.20, 8.26 ± 4.14, and 2.15 ± 1.83 g/d and corresponding N retention of 1.94 ± 0.35, 1.32 ± 0.66, and 0.34 ± 0.29 g/d for ad libitum, 30% feed restriction, and 60% feed restriction, respectively. This range, spanning from positive to near-zero protein balance, provided the variation necessary for the regressions used to derive NPm and MPm (Equations 3 and 4).

The NPm was estimated from the intercept (−0.282 g/kg BW^0.75^ per day) of the regression between retained N and CP intake (Equation 3), resulting in 1.76 g/kg BW^0.75^ per day:

[3] Retained N (g/kg BW^0.75^ per day) = −0.282 (±0.125) + 0.095 (±0.015) × CP intake (g/kg BW^0.75^ per day).

The NPm estimated in this study falls within the range of values reported for growing goats in the literature. Estimates of 1.39 g/kg BW^0.75^ (Ferreira et al., 2015) and 1.46 g/kg BW^0.75^ (Almeida et al., 2015) have been reported for growing Saanen goats older than 6 mo, whereas higher values were observed in younger or more heterogeneous populations, including 2.43 g/kg BW^0.75^ for F_1_ Boer × Saanen goats between 5 and 25 kg BW (Teixeira et al., 2017) and 2.12 g/kg EBW^0.75^ across dairy, meat, and indigenous breeds covering 4.5 to 51 kg BW (Almeida et al., 2020). These differences likely reflect variation in age, BW range, genotype, and physiological stage, all of which influence metabolic activity, protein turnover, and maintenance requirements.

The MPm estimated from regression of retained protein on MPI (Equation 4) was 2.50 g/kg BW^0.75^ per day when retained protein was set to zero (Figure 1):

[4] Retained protein (g/kg BW^0.75^ per day) = −0.595 (±0.172)  + 0.239 (±0.038) × MPI (g/kg BW^0.75^ per day).

The MPm estimate obtained in the present study falls within the range proposed by feeding systems, including 2.19 g/kg BW^0.75^ (AFRC, 1998), 2.52 g/kg BW^0.75^ (INRA, 2019), and 3.07 g/kg BW^0.75^ (NRC, 2007). However, it was notably lower than the estimates of 3.8 g/kg BW^0.75^ and 4.4 g/kg BW^0.75^ reported by Almeida et al. (2020) and Teixeira et al. (2017), respectively. These differences may reflect variation in dietary protein digestibility, diet composition, and assumptions regarding MCP synthesis and RUP digestibility, which directly influence estimates of MP utilization efficiency.

**Figure 1:**
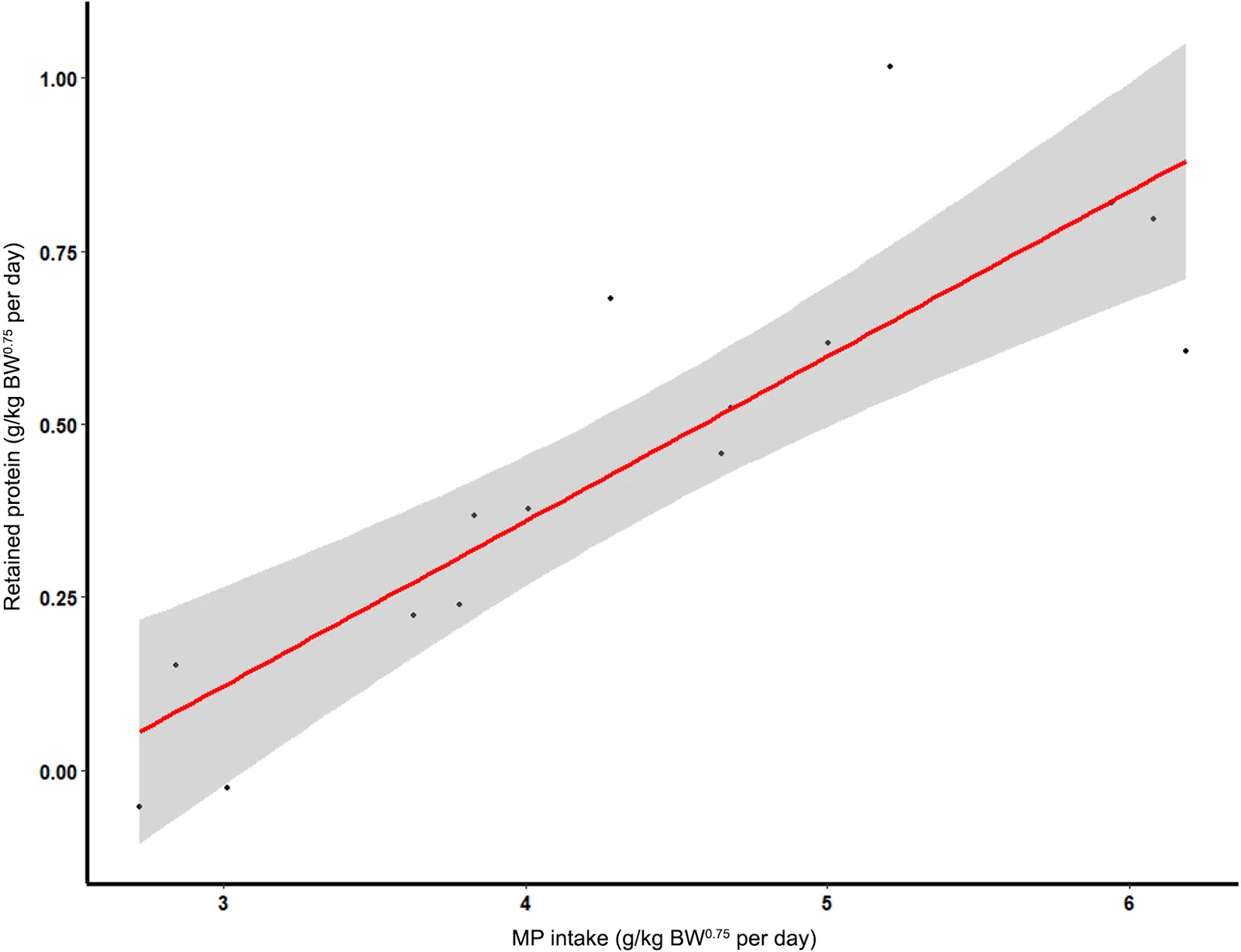
Relationship between retained protein (g/kg BW^0.75^ per day) and metabolizable protein intake (MPI, g/kg BW^0.75^ per day) in growing female Saanen goats. Protein retained = −0.595 (±0.172) + 0.239 (±0.038) × MPI. *P* = 0.0002; R^2^ = 0.76. The shaded area represents the 95% CI of the fitted regression line.

The ratio NPm/MPm resulted in a K_PM_ of 0.71. This estimate is within the range of values proposed by feeding systems, including 0.67 for dairy cattle (NRC, 2001), 0.70 for cattle and sheep (CSIRO, 1990), and 0.75 for cattle and sheep (ARC, 1980). Our K_PM_ estimate was higher than the values of 0.33 (Souza et al., 2021) and 0.55 (Teixeira et al., 2017; Almeida et al., 2020).

Monte Carlo simulations indicated minimal variation in the uncertainty of NPm and MPm estimates in growing female goats across the evaluated BW range (30 to 45 kg BW; Table 1).

**Table 1:** Estimated net and metabolizable protein requirements for maintenance and growth of female Saanen goats between 30 and 45 kg BW^1^

Requirement	BW (kg)	Mean	SD	LCI	UCI
NPm^2^ (g/d)	30	22.53	10.05	3.00	42.29
	35	25.29	11.29	3.37	47.47
	40	27.95	12.48	3.72	52.47
	45	30.53	13.63	4.07	57.32
MPm^3^ (g/d)	30	31.32	4.98	19.72	39.06
	35	35.16	5.59	22.13	43.85
	40	38.86	6.18	24.46	48.47
	45	42.45	6.75	26.72	52.9
NPg^4^ (g/kg BW gain per day)	30	104.32	8.23	88.82	120.96
	35	104.48	8.98	87.67	122.96
	40	104.39	9.64	86.44	124.21
	45	103.84	10.18	84.82	124.82
MPg^5^ (g/kg BW gain per day)	30	396.73	43.83	316.81	492.29
	35	397.34	45.92	313.70	497.80
	40	396.97	47.75	309.77	501.94
	45	394.89	49.22	305.02	503.09

1 Monte Carlo simulations (10,000 iterations) were used to estimate distributions for each parameter. Means and SD were calculated from the simulated distributions, and the 95% CI (LCI and UCI) correspond to the 2.5th and 97.5th percentiles. LCI = lower 95% confidence limit; UCI = upper 95% confidence limit.

2 NPm = net protein requirement for maintenance, estimated from the intercept of retained N (g/kg BW^0.75^ per day) on CP intake (g/kg BW^0.75^ per day): Retained N = −0.282 (±0.125) + 0.095 (±0.015) × CP intake.

3 MPm = metabolizable protein requirement for maintenance, estimated from the regression of retained protein (g/kg BW^0.75^ per day) on MP intake (g/kg BW^0.75^ per day): Retained protein = −0.595 (±0.172) + 0.239 (±0.038) × MPI.

4 NPg = net protein requirement for growth, estimated from the first derivative of the allometric equation relating body protein and EBW and expressed relative to BW gain using BW/EBW conversion factors.

5 MPg = metabolizable protein requirement for growth, calculated from NPg and the efficiency of metabolizable protein utilization for growth (K_PG_).

Body protein composition was estimated using an allometric equation between log-transformed body protein and log-transformed EBW in female goats weighing from 30 to 45 kg BW (Equation 5; Figure 2):

[5] Log_10_Protein (g) = 2.425 (±0.075) + 0.832 (±0.051) × Log_10_EBW (kg)

The NPg (g/kg EBW gain), estimated from the first derivative of Equation 5 (Equation 6), decreased from 130.40 to 120.45 g/kg EBW gain as BW increased from 30 to 45 kg:

[6] NPg (g/kg EBW gain) = 221.835 × EBW^(−0.1677)^ (kg).

When expressed relative to BW gain, Monte Carlo simulations estimated NPg to decrease from 104.32 to 103.84 g/kg BW gain as BW increased from 30 to 45 kg. This gradual decline likely reflects changes in body composition during growth, with increasing adipose tissue accretion as animals approach maturity. Almeida et al. (2016) estimated mature EBW of female Saanen goats at 26 kg EBW, indicating that most animals evaluated in this study were approaching or had already surpassed this threshold (i.e., 23.9 to 38.8 kg EBW).

**Figure 2:**
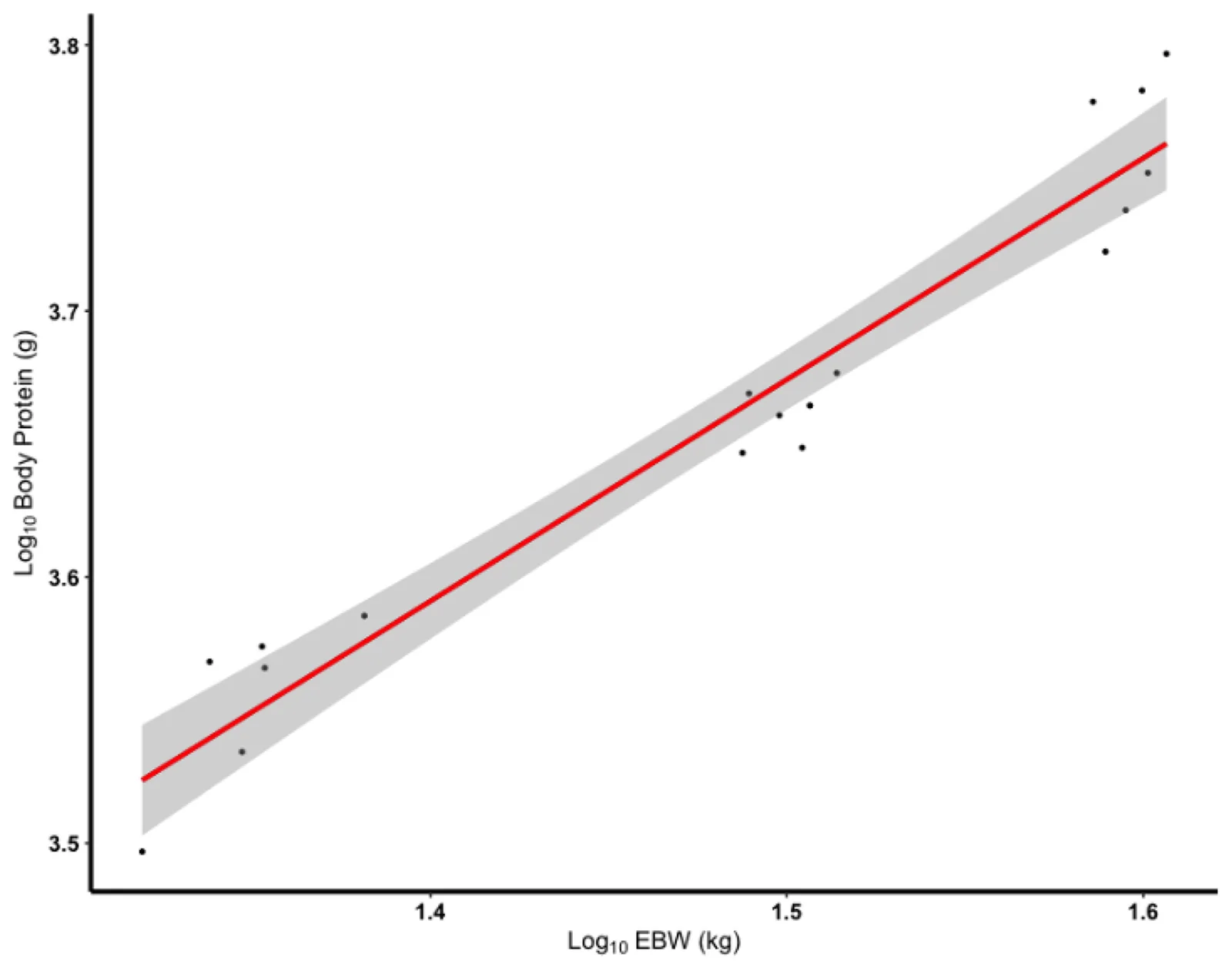
Relationship between log-transformed body protein (g) and log-transformed empty body weight (EBW; kg) in growing female Saanen goats. Log_10_Protein (g) = 2.425 (±0.075)  + 0.832 (±0.051) × Log_10_ EBW (kg). *P* < 0.0001; R^2^ = 0.94. The shaded area represents the 95% CI of the fitted regression line.

The NPg estimates obtained in this study were lower than those reported by Almeida et al. (2015; 132.4 to 130.4 g/kg BW gain), Ferreira et al. (2015; 140.7 to 135.9 g/kg BW gain), and Souza et al. (2017; 140 to 131 g/kg BW) for growing Saanen goats within similar BW ranges. The fact that all these studies reported a declining NPg with increasing BW supports the interpretation that changes in body composition are a major driver of this pattern. The smaller decline observed herein likely reflects the narrower and more physiologically homogeneous BW range evaluated, in which goats are in a more advanced stage of growth and progressively deposit adipose tissue relative to lean tissue.

The K_PG_, estimated from the relationship between daily retained protein and MPI above maintenance, was 0.26 (±0.0203), which was similar to values reported by Herbster et al. (2022; 0.25), but lower than the values proposed by AFRC (1992, 1998; 0.59) and Souza et al. (2021; 0.52). This relatively low K_PG_ is consistent with the late-growth stage of the animals evaluated in this study. As goats approached their mature weight, the composition of BW gain progressively shifts from lean tissue toward adipose tissue, reducing protein deposition while a greater proportion of gain is deposited as fat (Owens et al., 1993). Consequently, a smaller proportion of MP available for growth is retained as body protein, resulting in lower K_PG_ values. The MPg was obtained by dividing NPg by K_PG_. Using Monte Carlo simulations, MPg ranged from 396.73 to 394.89 g/kg BW gain as BW increased from 30 to 45 kg (Table 1). Monte Carlo simulations indicated minimal variation in the uncertainty of MPg estimates across the evaluated BW range.

Our MPg estimates exceeded the 290 g/kg BW gain reported by NRC (2007), and were also higher than those proposed by INRA (1989; 360 g/kg BW gain), AFRC (1998; 240 g/kg BW gain), and Luo et al. (2004; 285 g/kg BW gain). These values likely reflect changes in body composition as animals approach maturity, with increasing adipose tissue deposition and reduced protein accretion per unit of gain, resulting in lower efficiency of dietary protein utilization for growth (Owens et al., 1993).

This study estimated MPm and MPg in growing female Saanen goats between 30 and 45 kg BW using the comparative slaughter technique. The estimated MPm is 2.50 g/kg BW^0.75^, whereas MPg ranges from 396.73 to 394.89 g/kg BW gain as BW increases from 30 to 45 kg. Our findings refine current protein requirements estimates for female dairy goats within a physiological phase and sex that remain underrepresented in the literature and support the development of more precise feeding strategies aimed at improving N utilization efficiency in dairy goat production systems.
